# Comparison of Serum Metabolite Changes of Radiated Mice Administered with *Panax quinquefolium* from Different Cultivation Regions Using UPLC-Q/TOF-MS Based Metabolomic Approach

**DOI:** 10.3390/molecules23051014

**Published:** 2018-04-26

**Authors:** Zhenxian Qin, Chan Jia, Dengqun Liao, Xiaofang Chen, Xian’en Li

**Affiliations:** Institute of Medicinal Plant Development, Chinese Academy of Medical Sciences & Peking Union Medical College, Beijing 100193, China; qinzhenxian@126.com (Z.Q.); jiachan0393@163.com (C.J.); dqliao@implad.ac.cn (D.L.); cpuxbl@126.com (X.C.)

**Keywords:** *Panax quinquefolium*, cultivation region, cobalt radiation, serum metabolomics, UPLC/Q-TOF-MS

## Abstract

Chemometric analysis of bioactive compounds revealed that American ginsengs (AGs) from different cultivation regions of China had a difference in quality, which indicates their possible pharmacological difference. A UPLC-Q/TOF-MS-based untargeted metabolomic approach was used to uncover serum metabolite changes in radiated mice pre-administered with AG root decoctions from seven cultivation regions and to further assess their quality difference. OPLS-DA revealed that 51 metabolites (ESI^−^) and 110 (ESI^+^) were differentially expressed in sera between the control and the radiated model mice. Heatmap analysis further revealed that AG could not reverse most of these radiation-altered metabolites, which indicates dietary supplement of AG before cobalt radiation had the weak potential to mediate serum metabolites that were altered by the sub-lethal high dose radiation. In addition, 83 (ESI^−^) and 244 (ESI^+^) AG altered metabolites were detected in radiated mice under radiation exposure. Both OPLS-DA on serum metabolomes and heatmap analysis on discriminant metabolites showed that AGs from different cultivation regions differentially influenced metabolic alterations in radiated mice, which indicates AGs from different cultivation regions showed the pharmacological difference in modulation of metabolite changes. AGs from Shandong, Shanxi, and Beijing provinces had more similar pharmacological effects than AGs from USA, Canada, Jilin, and Heilongjiang. Finally, 28 important potential biomarkers were annotated and assigned onto three metabolic pathways including lipid, amino acid, and energy metabolisms.

## 1. Introduction

American ginseng (*Panax quinquefolius* L., Xi yangshen in Chinese) is a perennial medicinal plant of the genus *Panax* in Araliaceae family. It has been a popular traditional Chinese medicine since the 17th century. Many studies demonstrated that American ginseng had a wide range of pharmacological effects including immunomodulatory [1,2,3], antioxidative [4,5,6], anti-inflammatory [7,8], anticancer [7,9,10], antidiabetic [11,12], and anti-aging effects [13]. Due to its high medical values like other ginseng species, AG roots known as the main used medicinal organs of this species are widely used in the world not only as drugs but also as dietary supplements and food. China is one of a large AG-consuming country and also one of three primary AG-producing countries including its places of origin, which is America and Canada. After the successful introduction into China in the 1980s, American ginseng is now cultivated in three main regions including Northeast China, Shandong Peninsula, and the Huairou district of Beijing and also on a small scale in other provinces such as the Qinling region in Shanxi [14,15]. AG products from the same or different cultivation regions of China or from native countries (USA and Canada) vary greatly in quality and root appearance, which usually influences their market share and pricing. In order to reveal the quality difference of AGs from different cultivation regions of China and to see whether our domestic AGs, after near 40 years of domestication, had a close quality to cultivated AGs of USA and Canada, Chinese researchers/scientists evaluated AG quality of different geographical origins based on the contents of saponins, which are the major bioactive compounds [14,15,16,17,18,19,20,21]. These results showed that AGs from the specific cultivation regions of China had a similar total saponin content and varied in content of some ginsenosides. Fourier infrared spectroscopy analysis on methanol-dissolved AG extracts uncovered the metabolite difference of AG products from seven cultivation regions including the United States, Canada, and five provinces of China (Heilongjiang, Jilin, Shandong, Shanxi, and Beijing) [22]. TCM theory thinks that therapeutic or pharmacological effects of an herbal medicine are determined by the comprehensive actions of all the metabolites within itself. In this sense, clinical therapeutic effects are more convincing and direct indicators for quality evaluation of medicinal plants. However, no such pharmacological research has been conducted until now to reveal whether AGs from different cultivation regions have different or similar medical effects and to further assess their quality difference.

Metabolomics, which is a relatively new branch, has become a powerful approach to qualitative and quantitative analysis of the entire metabolites (mass < 1000 Da) within a given sample, which provides the metabolite fingerprinting of the sample and, therefore, could distinguish multiple samples under different physiological statuses or could be subjected to an environmental stimulus. Many high-throughput analytical techniques including NMR spectroscopy, LC-MS, and GC-MS are applied in metabolomics studies because of their efficiency in separating metabolites and identifying patterns or metabolite markers among a great number of samples. Pharmacology-based metabolomics has the potential not only to study pharmacological effects of herbal plants and their metabolic action mechanisms [23,24] but also to discriminate plant species of the same genus [25,26] and the same plant species from different cultivation regions [27]. It was also adopted to assess the holistic quality of herbal medicinal products [28].

Ionizing radiation occurs accidentally or regularly around us, which renders various health problems. ^60^Co-γ radiation is often applied to study radiation-induced injuries on living organisms and radiation-therapy effects and to screen radiation-protective or alleviating agents [29,30,31,32,33]. ^60^Co-γ radiation generates reactive oxygen species (ROS) and results in many adverse biological effects including the damage of DNA, proteins, and lipids at the molecular level and consequent changes of gene expression and blood biochemicals or metabolites, which further injures hematopoietic, immunological, and gastrointestinal tissues. This depends on the radiation dose and dose rate adopted in the experiments. The genome-wide gene expression analysis revealed that radiation exposure mainly induced the genes involved in the immune system and in cancer development pathways. 1 Gy radioactive exposure was likely the critical threshold dosage for carcinogenesis and metabolic disorders in peripheral blood mononuclear cells [34]. Acute phase protein SAA serum amyloid A (SAA), which is an indicator of the severity of acute radiation syndrome (ARS), peaked one day after cobalt radiation and was independent on the radiation dose [32]. The dual biomarkers of total-body irradiation known as the IL-18 binding protein (IL-18BP) and IL-18 were both increased in mice serum on Day 1 after 5 Gy doses of cobalt radiation [35]. These studies indicated that cobalt radiation could induce the rapid changes of serum biochemical components within a few hours or one day after radiation.

Due to its updated and circulating characteristics and no damage to living organisms, serum is often collected for disease diagnosis and therapy effects of medicines through assays of enzyme or metabolite biomarkers inside itself. LC-MS or NMR-based metabolomic analysis revealed that metabolites belonging to amino acids, fatty acids, and lipids were altered in sera of radiated mice or radio-therapeutic patients [36,37,38,39]. Therefore, a UPLC-Q/TOF-MS-based untargeted metabolomics combining multivariate analyses was employed to investigate the holistic metabolite alterations in murine serum one day after cumulative 5.0 Gy of ^60^Co γ-ray radiation. The Ex Vivo ^137^Cs γ-ray radiation experiment showed that North American ginseng extract (NAGE) had the radio-protective effects on human lymphocytes through the increase of total antioxidant capacity (TAC) and decrease of the reactive oxygen species (ROS) before and after ^137^Cs-radiation [40,41]. As a popular herbal antioxidant, American ginseng is taken not only as a regular dietary supplement but is often used in many cancer therapies [42]. Sometimes, radiation is needed for cancer therapy or illness diagnosis. In some cases, American ginseng was used with other drugs or Chinese herbals to improve cancer-related symptoms during or post radiotherapy of cancers [43,44,45,46,47]. Therefore, we investigated the potential modulating effects of AG pre-administration on radiation-induced metabolite alterations in serum of radiated mice and compared the pharmacological difference of AGs from seven cultivation regions, which will facilitate the overall quality assessment and control of AGs.

## 2. Results

### 2.1. Method Validation

**Figure 1:**
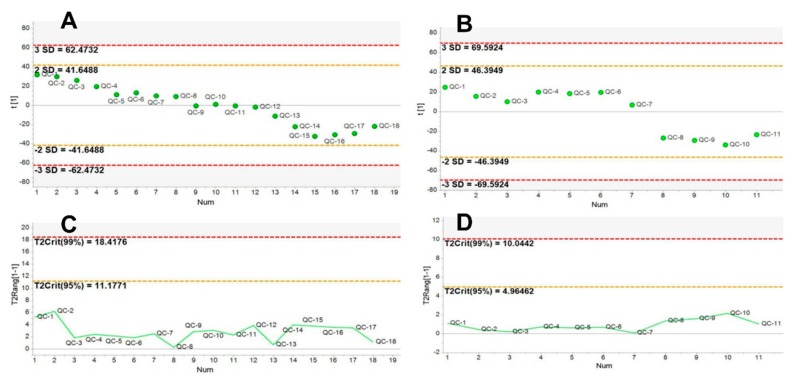
Line plots showing PCA of different injections of the pooled QC sample. The X-axis represented run order of the QC sample, the Y-axis represented standard deviation (**A**,**B**), and Hotelling’s T2 range (**C**,**D**), separately. (**A**,**C**) positive ion mode and (**B**,**D**) negative ion mode.

The typical chromatograms of serum samples in both positive and negative ion modes were shown in Figure S1A,B, respectively. As shown in Figure S1, the metabolites could be well separated within 15 min using the optimized UPLC/Q-TOF-MS conditions. In order to monitor and access the chromatographic stability and reproducibility, repetitive injections of the same quality control (QC) sample were performed before and during sample analysis. Four abundant ion peaks (1.13_226.9511, 3.73_517.9415, 8.73_496.3408, and 9.06_522.3571) were extracted from the positive ion chromatograms of QC samples for method validation. The precision of injection was validated by analysis of six continuous injections of the same QC sample. The RSDs ranged from 0.11% to 0.54% for the retention time and from 5% to 16.7% for peak intensity. Afterward, method repeatability was validated by six replicate analysis of the QC sample. The RSDs of retention time and peak intensity were estimated to be 0.05% to 1.7% and 5.9% to 13.5%, separately. We further evaluated the deviation variation of all the QC samples via principal component analysis (18 QC samples in the positive ion mode and 11 in the negative ion mode). It can be seen that all the QC samples fell within the 2SD’s region and the plot of 95% confidence interval (see Figure 1). All these data indicated that our experimental conditions were suitable for metabolomic data analysis of experimental samples.

### 2.2. Cobalt-Radiation Induced Metabolite Changes in Mouse Serum

**Figure 2:**
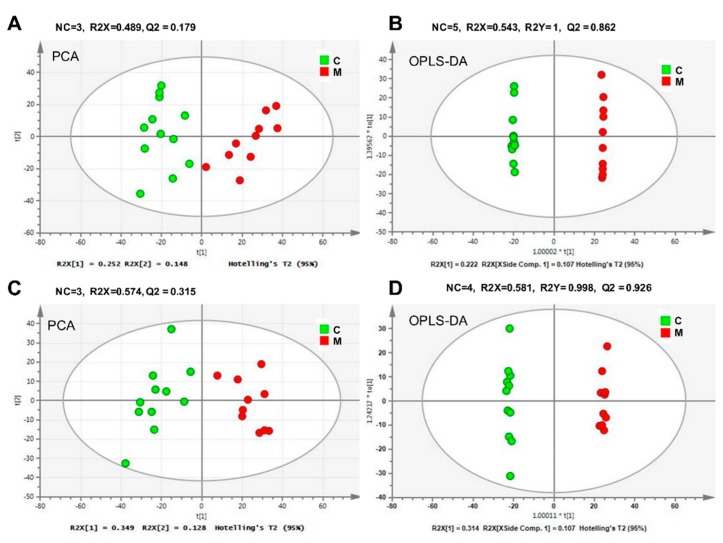
PCA and OPLS-DA score plots based on UPLC-Q/TOF-MS profiling of serum metabolomes from control group (C) and model group (M). (**A**,**B**) for ESI^+^ mode, (**C**,**D**) for ESI^−^ mode. NC meant number of components. R2X, R2Y, and Q2 were cumulative fractions of all the R2Xs, R2Ys, and Q2, respectively. P_CV-ANOVA_ values for OPLS-DA models (**B**,**D**) were separately, 0.000602037 and 1.16212e-005.

Metabolomic profiling of mouse serum samples yielded a total of 3653 peaks in the positive ion mode and 809 peaks in the negative ion mode, respectively. Principal component analysis (PCA) and Orthogonal partial least squares discriminant analysis (OPLS-DA) are the two most widely used methods in multivariate analysis of high dimensional metabolomic data [48,49]. As an unsupervised multivariate statistic method, PCA depicts the general relationship of experimental samples. OPLS-DA is a popular and effective supervised multivariate analysis, which separates the systematic variation in X into between-class variation (Y-linear) and is uncorrelated within-class variation (Y-orthogonal), which makes metabolomic data more easy to interpret [50]. In our study, we first performed PCA and OPLS-DA to discern whether the cumulative 5.0 Gy of ^60^Co γ-ray radiation caused metabolite change in mouse serum. Both analyses revealed that there was a complete separation between the model and control groups (see Figure 2). In the positive ion mode, the PCA resulted in three principal components with R2X (cum) = 0.489 and Q2 (cum)  = 0.189. OPLS-DA resulted in one predictive component and four orthogonal components. The predictive component (R2X1 = 0.222) could reveal all the variance in the class separation (R2Y cum) with the predictive ability of 86.2% (Q2 cum). In the negative ion mode, PCA resulted in three principal components, which revealed 48.9% of the total variance induced by radiation exposure. OPLS-DA obtained one predictive component with R2X = 0.314 and three orthogonal components, which explained 99.8% of the variations among all the samples. The predictive ability of Q2 (cum) was 92.6%. At the same time, the robust of two constructed OPLS-DA models was further confirmed by Coefficient variability analysis of variance (CV-ANOVA) with a *p* value of 0.000602037 and 1.16212e-005, respectively. Therefore, the cobalt-radiated model was successfully established and can be further used for the pharmacological analysis of American Ginseng on ionizing radiation and for quality assessment of AGs from different cultivation regions. However, individuals within the same treatment group were dispersed in the unsupervised PCA score plots and divided into two major subgroups in OPLS-DA score plots, which indicated that the contents of serum metabolites varied greatly among individuals under the same treatment.

**Figure 3:**
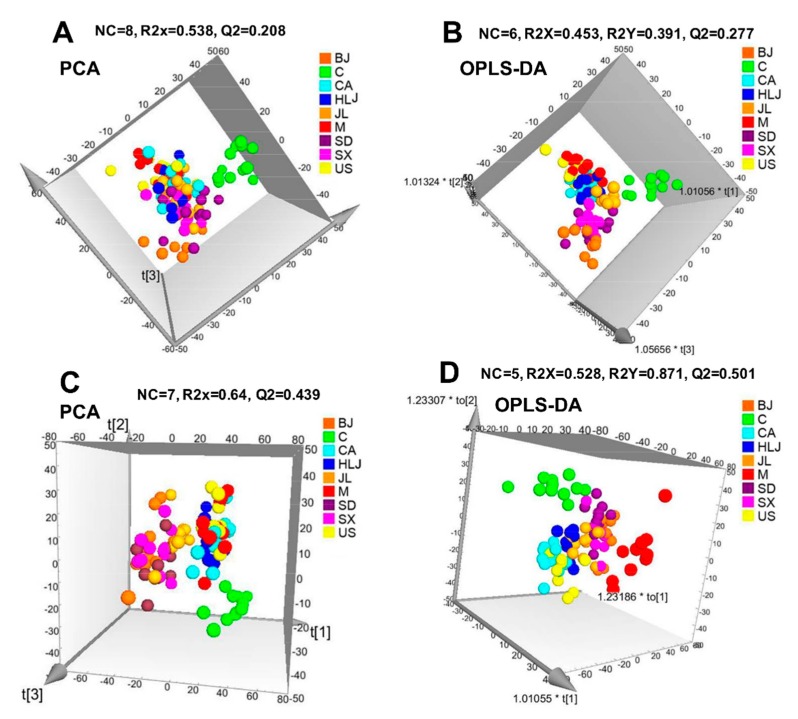
PCA and OPLS-DA score plots of serum metabolomes from control group (C), Co-radiated model group (M) and AG groups. ESI^+^ mode: (**A**,**B**); ESI^−^ mode: (**C**,**D**). NC meant number of components. R2X, R2Y, and Q2 were cumulative fraction of all the R2Xs, R2Ys, and Q2, respectively. P_CV-ANOVA_ values for OPLS-DA models (**C**,**D**) were separately, 4.74629e-013 and 2.35445e-009.

In order to reveal whether pre-administration of AG decoction could modulate radiation-induced metabolite alterations in the sera of radiated mice, we subsequently performed PCA and OPLS-DA on metabolomic data of all the nine groups (see Figure 3). In the positive ion mode, the PCA and OPLS-DA results (see Figure 3A,B) showed that the non-irradiated normal control group was separated from the model and AG groups (for the convenience, AG group(s) here and after was referred to AG-pre-administered radiated group(s)). However, there was no apparent separation between AG groups and the model group. The OPLS-DA model produced four predictive components and two orthogonal components. The quality parameters of OPLS-DA model were R2X (cum)  = 0.453, R2Y (cum)  =  0.391, Q2 (cum)  =  0.277, separately. In the negative ion mode, OPLS-DA fitted mode resulted in one predictive and four orthogonal components with R2X (cum)  = 0.528, R2Y (cum) =  0.871, Q2 (cum)  = 0.501 (see Figure 3D). The model control group was separated from both the control group and AG groups, which indicates that the negative ion mode was more powerful to discriminate different AG treatments than the positive ion mode. *p* values obtained from CV-ANOVA validation also showed that the OPLS-DA models constructed by nine groups were statistically significant and workable.

### 2.3. Serum Metabolite Changes in Radiated-Mice Administered with AG Decoction

In pairwise comparisons of the model group and the individual AG groups, OPLS-DA discriminated all AG-administered mice groups from the mouse model (data not shown). OPLS-DA comparing the model group, the control group, and each individual AG groups showed that AG-pretreated groups especially in the negative ion mode were separated from the cobalt-radiated model group and also from the control (see Figure S2A,B), which suggests that the dietary supplement of American ginseng can interrupt the serum metabolism under normal and adverse conditions. Although Figure 3 could not reveal that all the AG groups were clearly separated from the model group, it was observed that some AG groups including US, CA, HLJ, and JL groups were much closer to the model group, which indicates that there was the pharmacologically radiation-responding difference of mice administered with AGs from the different cultivation regions.

**Figure 4:**
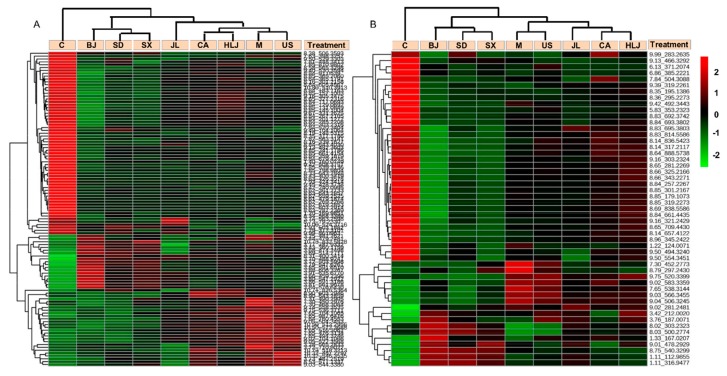
Hierarchical clustering analysis of cobalt-altered metabolites in sera of the control (C), model (M), and AG mice. The heatmaps were generated in MetaboAnalyst using the differential biomarkers identified between the model group and the control group. ESI^+^ mode: (**A**); ESI^−^ mode: (**B**).

In order to reveal whether pre-administration of AG decoction could have anti-radiation effects on frequent or occasional exposure to ionizing radiation at the metabolite level, we first investigated the influence of AG pretreatment on cobalt-radiation altered metabolites, which were identified in OPLS-DA models by comparing the model and control groups (Table 1, Figure 4A,B). In the positive ion mode, Table 1 showed that a total of 110 metabolites (46 increased/64 decreased) were altered between the control and radiated model group, which was based on our differential metabolite screening criteria described in the method. In total, 68 common metabolites were found significantly distinct between the control and all AG groups. In the negative ion mode, 51 metabolites with 15 metabolites increased and 36 metabolites decreased in the model mice, which were altered by cobalt radiation compared to the control group. Heatmap analysis on these differential metabolites revealed that they had similar changing trends in sera of AG-administered mice groups as the model group (see Figure 4A,B), which suggests that the supplement of AG decoction before cobalt radiation could not modulate the radiation-altered metabolites especially in our experiment where cobalt radiation was applied with a high dose within the short time. At the same time, our heatmap results also revealed that mice groups receiving AG decoctions of different cultivation regions were clustered into two major groups. The cluster containing US, CA, HLJ, and JL groups had less differential metabolites from the model group. Their metabolite contents were closer to those of the model group. The second sub-cluster comprising SD, SX, and BJ groups had more differential metabolites relative to the model group. The changing values of the differential metabolites in sera of these three AG-treated mice were higher than those of the former four AG groups.

**Figure 5:**
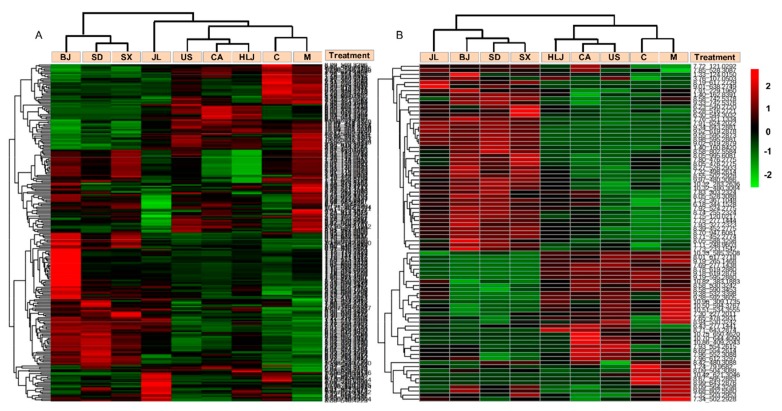
Hierarchical clustering analysis of AG-induced differential metabolites among different treatments. The heatmaps were generated in MetaboAnalyst using the differential biomarkers of importance identified between the model group and various AG groups. The biomarkers that were identified between the control and model groups were excluded in this analysis. ESI^+^ mode: (**A**); ESI^−^ mode: (**B**).

In order to identify AG altered metabolites under the condition of radiation exposure, we made pairwise comparisons of individual AG groups and the model group (Table 1, Figure 5A,B). In the positive ion mode, a total of 291 metabolites were significantly changed between AG groups and the model radiated group, which included 47 metabolites differentially expressed between the control and model groups. The number of significantly changed metabolites between AG groups and the model group ranged from 17 (USA) to 146 (BJ), which indicates that AGs from the different cultivation regions affected mouse serum metaboliomics differently when exposed to cobalt radiation. In the negative ion mode, a total of 111 metabolites were significantly altered by pre-administration of AG decoction, which included 28 metabolites differentially expressed between the control and model groups. Additionally, 13 (USA) to 57 (BJ) differential metabolites were detected between AG groups and the model group. Heatmap analysis on these AG altered metabolites (see Figure 5A,B) observed similar results as Figure 4A,B with regard to the influence of place origins of AG decoctions on metabolite changes in radiated mouse serum. Therefore, AGs from different cultivation regions showed the pharmacological difference in modulation of metabolite changes. SD, SX, and BJ groups were still clustered together and showed different metabolite alterations from US, CA, and HLJ groups, which were located on the same branch as the model and control groups. The model and control groups were clustered on the same branch than the other seven AG groups, which reflects that some metabolites in the mouse serum were induced specifically by AG pre-administration (see Figure 5A,B).

### 2.4. Biomarker Identification and Metabolic Pathway Analysis

**Figure 6:**
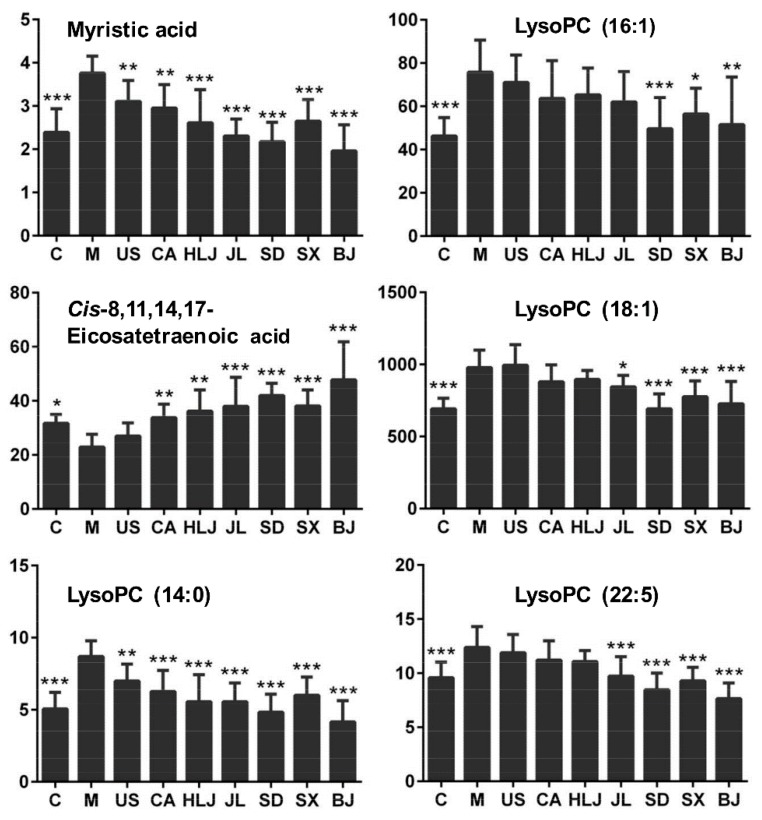
Peak intensities of six representative potential biomarkers in serum of each group. Y-axis showing peak intensity was indicated by peak area. * *p* < 0.05, ** *p* < 0.01, and *** *p* < 0.001 meant the significant difference of metabolites in the control and AG groups compared with the model group. These six biomarkers were significantly induced by cobalt radiation and modulated to various degrees by AG decoctions from different cultivation regions.

Among the statistically significant differential metabolites with VIP values ≥1, 28 metabolites were annotated as fatty acids, amino acids, and lysophospholipids and assigned onto three KEGG metabolic pathways including lipid, amino acid, and energy metabolism (see Table 2). As such, 20 metabolites were found to have the fragment ions compared with the reported characteristic ions in research studies (see Table S1). Additionally, 22 of these identified metabolites showed the differential expression between the control and radiated model groups of which six lipid metabolites including myristic acid, *cis*-8,11,14,17-eicosatetraenoic acid, LysoPC (14:0), LysoPC (16:1), LysoPC (18:1), and LysoPC (22:5) were attenuated or increased by AG decoction especially from Shandong, Shanxi, Jilin, and Beijing provinces (see Figure 6). Lipid peroxidation was increased greatly in the radiated mouse and can be reduced by antioxidants such as melatonin [29]. As a satisfactory antioxidant, American ginseng may be related to the reduction of lipid peroxidation, which increased the contents of unsaturated fatty acids and several lysophospholipids.

## 3. Discussion

In this study, we aimed to investigate the in vitro pharmacological effect of American ginseng on radiation via an untargeted serum metabolomic analysis and to reveal the quality difference of American ginseng from different cultivation regions. Although ex vivo experiments revealed that American ginseng had the radioprotective effects in reducing radiation-induced DNA damage through the increase of the total oxidant and the reduction of ROS [40,41], our serum metabolomic results showed that pre-treatment of American ginseng could not reverse most of the radiated-altered serum metabolites in mice with only a few metabolites ameliorated. This result indicated that American ginseng had the very weak radiation-protective or therapeutic effects in terms of modulating the overall radiation-altered metabolites in the serum. It was observed that the radio-protective effect of AG on human lymphocytes was concentration-dependent, dose-dependent, and administration-time-dependent [40,41]. In these two ex vivo studies, AG was administered one day before or 90 min after radiation exposure. However, our serum samples were collected for the metabolomic analysis six days after the last administration, which consisted of five days of consecutive radiation. In addition, the high-dose radiation we used in this experiment is a sub-lethal dose that caused irreversible injuries on living organisms [29]. Therefore, the radiation-induced metabolite changes were unable to be reversed by AG in our experimental conditions. However, our present finding of AG modulation on radiation-induced metabolite changes need to be further validated with a relatively low-dose and short-term radiation. In addition, observation should be made around the last administration of AG decoction. Furthermore, the continued duration of AG in vivo radioprotective effects also should be investigated. These future studies combining our current result will provide some more information on the potential radioprotection of American ginseng and the optimal administration time during radiation therapy or diagnosis.

In addition, our results could still reveal that there was a pharmacological effect difference in American ginseng from different cultivation regions in this radiation experiment. AGs grown in Shandong, Shanxi, and Beijing provinces showed more similar pharmacological effects than AGs from four other cultivation regions including USA, Canada, Jilin, and Heilongjiang. Zhang et al. [51] found that AG quality, which was assessed by ginsenoside content, was more highly correlated with its origin place than traditional commercial specifications (root size and length). American ginseng originated from the United States and Canada, which are now two important producing areas of American ginseng in the world. Based on ecotype data and ginsenoside content, Huang et al. [17] classified the domestic AGs into two major chemo-ecotype groups: Beijing and Shandong group and Northeast group including Heilongjiang, Jilin and Liaoning provinces. Using the infrared spectroscopy method, Jia et al. [22] classified the above seven AG groups into the US-group and the Shanxi-group, the Canada group and the third group containing AG from HLJ, BJ, SD, and JL provinces, which was somewhat different from our in vivo metabolomic results.

In summary, the long-term dietary supplement of AG had a weak potential to mediate the metabolite alterations that were induced by the sub-lethal high dose radiation. AGs from different cultivation regions showed the pharmacological difference in modulation of metabolite changes under radiation. In Vitro experiments and AG utilization in cancer radiotherapy proved that AG had the radioprotection or improvement on radiation side effects. Our current result combines future studies such as the preventive efficacy of AG and its lasting time as a potential radioprotector. This will provide some more information on its optimal administration dose/time during radiation therapy or diagnosis.

## 4. Materials and Methods

### 4.1. American Ginseng Root Samples and Decoction Preparation

A total of 38 four-year-old AG main root samples were used to study radiation-related pharmacological effects of American ginseng from seven different geographical locations including the United States (represented by Wisconsin state), Canada (represented by Quebec province), and five provinces of China including Heilongjiang, Jilin, Shandong, Shanxi, and Beijing. Among these samples, 25 domestic American ginseng root samples were collected from farms in 2015 and 11 imported root samples were purchased from Beijing Tongrentang Co., Ltd., Beijing, China. Another two root samples were obtained directly from American provinces (Wisconsin state) and Canada (Quebec province). These 38 dried root samples were separately ground and sieved via a 60 mesh sieve. Then the equal amount of root powders from different batches (cultivation regions) from the same originating province/country were weighed and mixed up, which served as the sample from the same province of China, USA, or Canada. Additionally, 20 grams of the mixed root powder in each province or country was immersed in 500 mL of distilled water for one hour and then boiled for 45 min. The extracted solution was filtered and the residue was re-extracted twice in the same way. Finally, three filtrates were combined, concentrated to 100 mL, and stored at a low temperature for the below administration experiment.

### 4.2. Animals, Administration, and Co_60_ γ-Radiation Experiments

The animal experiments were performed according to the Guidelines of National Health Institutes of China for the Care and Use of Laboratory Animals and approved by the Institutional Animal Care and Welfare Committee of IMPLAD (Institute of Medicinal Plant Development) with the certificate No. SYXK2013-0023 (Jing). Additionally, 108 five-week old male ICR mice (18–22 g) were purchased from the Peking University Health Science Center, Beijing, China (No. SCXK (Jing) 2014–0006) and housed at the temperature of 22 °C to 23 °C and humidity of 50% to 60% in a 12/12 h light/dark cycle at the animal center of IMPLAD, Beijing, China. All the mice could freely access an aseptic diet and tap water. After one week of acclimation, 108 mice were randomly divided into nine equal groups, which includes the control (vehicle) group, the model control group, and seven groups that were administered separately with AG decoctions of seven cultivation regions. For the convenience, the administered mice group was named after the production province or country of its administered AG decoction including the US, CA, HLJ, JL, SD, SX, and BJ group. Additionally, 0.2 mL AG decoction /10 g mouse weight was intragastrically injected into each mouse daily for four consecutive weeks. At the same time, the mice in the control and model groups received an equal volume of distilled water. After the last administration, mice in model and administered groups were whole body irradiated for five consecutive days with the daily 1.0 Gy ^60^Cobalt γ-rays.

### 4.3. Serum Metabolomic Analysis

#### 4.3.1. Serum Sample and QC Sample Preparation

In total, eight to 12 mice in each group were used to study radiation-associated pharmacological effects of American ginseng from seven different geographical locations by using mouse serum metabolomic analysis. The day after the last ^60^Cobalt-γ radiation, blood samples were collected through eye vessels by removing mouse eyeballs and then centrifuging at 4 °C at 3500 rpm for 10 min to isolate serum supernatant for serum metabolomic analysis. They were immediately stored in −80 °C until undergoing UPLC-Q/TOF-MS analysis. Prior to the analyses, the frozen serum samples thawed at 4 °C. 200 μL of the serum sample was mixed with 600 μL of 4 °C pre-cooled acetonitrile (ACN) and vortexed for 30 s. The mixture was kept on ice for 15 min and subsequently centrifuged at 15,000 rpm at 4 °C for 15 min. The supernatant was collected, filtered through a 0.22 μm membrane filter, and then transferred into the inner vial in order to undergo UPLC/Q-TOF-MS analysis. Meanwhile, 10 μL of the individual serum supernatant samples from the different groups were taken and mixed up as the quality control (QC) sample. The steps to prepare the QC sample for UPLC/Q-TOF-MS analysis were performed on the aforementioned serum samples.

#### 4.3.2. UPLC/Q-TOF-MS Analysis

Five microliters of the serum sample were separated at 40 °C on a Waters Acquity^TM^ Ultra Performance LC system (Waters Corporation, Milford, MA, USA) and coupled with a Waters UPLC BEH C18 column (1.7 µm, 2.1 × 100 mm, Waters Corp. Milford, MA, USA). The flow rate was set to 0.2 mL/min. The mobile phase consisted of 0.1% formic acid in water (A) and ACN (B) and was run in an optimized gradient program using the following parameters: 5% B at 0–0.5 min, 5–30% B at 0.5–2 min, 30–60% B at 2–5 min, 60–80% B at 5–8 min, 80–100% B at 8–11 min, 100–5% B at 11–13 min and 5% of B for 2 min to re-equilibrate the column. All the samples of the same group were analyzed before the next group of samples was initiated. Seven consecutive injections of the QC sample were performed to stabilize the instrument system before the formal samples were analyzed. At the same time, in order to monitor the condition and stability of the UPLC instrument system, the blank and the abovementioned QC sample were injected recurrently during the run of each group.

MS data acquisition was performed in the positive/negative ion modes on the Synapt G2 high definition mass spectrometer system (Waters Corporation, Milford, MA, USA), which was equipped with an ESI ion source. The optimized ESI conditions included capillary voltage, 3 kV for the positive ion mode, 2.8 kV for the negative ion mode, a sampling cone voltage of 40 V, a desolvation gas temperature of 450 °C with a desolvation gas flow of 600 L/h, a source temperature of 120 °C with a cone gas flow of 50 L/h, and collision energy of 6 V. The centroid data were acquired over the range of *m*/*z* 50–1000 at a scan rate of 0.15 s with the inter-scan delay of 0.02 s.

#### 4.3.3. Multivariate Analysis of UPLC/Q-TOF-MS Data

The raw chromatographic data were then introduced into the MarkerLynx Applications Manager package of MassLynx 4.1 software (Waters Corp., Manchester, UK) for peak extraction and assignment. The parameters for peak extraction and alignment included a retention time range of 0–15 min, an XIC window of 0.02 Da, peak width at 5% height and 1 s, default peak–peak baseline noise, smoothing applied, marker intensity threshold (counts) of 100, a mass tolerance of 0.02 Da, RT windows of 0.1 s, a noise elimination level of 6, and deisotope peaks selected. The resultant data including retention times, *m*/*z*, and ion intensities were exported as USP files and then transferred into SIMCA-P software (V.13.0, Umetric, Umea, Sweden) for pareto normalization, multivariate analysis, and identification of differentially-expressed metabolites between two compared groups of mice. Multivariate analyses (MVA) including PCA and OPLS-DA were used to discern the global metabolomic differences among the control (vehicle) group, the model control group, and administered groups. The quality of OPLS-DA models was evaluated by *p*-values obtained in CV-ANOVA analysis. The default 7-round cross-validation was applied during the construction of MVA models and CV-ANOVA analysis.

To identify potential biomarkers for radiation-induced injuries and screen potential biomarkers contributing to anti-radiation effects of AG decoction as well as explain differential anti-radiation effects of AGs from different cultivation regions, OPLS-DA models between two groups were established for pairwise comparisons. The differential biomarkers were obtained through the following three sequential screening steps: (1) to select the variables with VIP ≥ 1 to construct S-plot for the list analyzed in Step 2, (2) to extract the former variables with |P(corr)| value ≥ 0.65 to construct a coefficient plot for the list analyzed in Step 3, and (3) to create the list in Step 2 whose Jack-knifed confidence intervals (CIJF_jk_) were present on the same side of the X-axis in the coefficient plot. These candidate biomarkers were further subjected to a two-tailed *t*-test to determine the statistical significance of the differential metabolites between two compared groups. To reveal the changing trends of the differential metabolites among different treated groups, these screened metabolites (ions) were further subjected to the unsupervised heatmap analysis, which was implemented by the MetaboAnalyst (http://www.metaboanalyst.ca/). The identities of significant biomarkers were deduced by comparing m/z information of their parent ion and MS/MS ion fragments with those of the Human Metabolome Database (http://www.hmdb.ca/), PubChem Compound databases (http://ncbi.nim.nih.gov/), and research reports. The mass error of deduced metabolites between the experimental mass and the theoretical mass was allowed to be ≤15 parts-per-million (ppm). The relevant metabolic pathways of deduced metabolites were obtained using the KEGG database (http://www.genome.jp/kegg/).

### 4.4. Metabolite Concentration

The concentrations of the detected metabolites in mouse serum were expressed as peak areas and their values for administered groups, model groups, and normal controls were reported as the means ± SD. One-way analysis of variance (ANOVA) followed by the Turkey’s multiple comparison test, which was applied using GraphPad Prism 6 software, was utilized to determine the statistical significance of the metabolites between the control and AG groups and the model group.

## Figures and Tables

**Table 1:** Number of increased/decreased metabolites revealed by pairwise comparisons between the model group and other groups.

ESI Mode	C	US	CA	HLJ	JL	SD	SX	BJ
+	46/64	6/11	19/28	21/48	39/59	37/43	31/54	71/75
−	15/36	7/6	13/10	13/8	18/11	20/19	26/22	27/30

**Table 2:** Identification of representative differential metabolites and their fold changes among different groups.

RT (min)	Quasi-Molecular Ion (*m/z*)	Cal. *m/z*	Mass Error (ppm)	HMDB ID	Metabolites	Formula	Metabolic Pathway	Fold Change							
								C-M	M-US	M-CA	M-HLJ	M-JL	M-SD	M-SX	M-BJ
* 7.30	468.3084 [M + H]^+^	468.3085	−0.140	HMDB10379	LysoPC (14:0)	C_22_H_46_NO_7_P	Lipid metabolism	1.72 ^c^	0.80 ^b^	0.72 ^c^	0.64 ^c^	0.64 ^c^	0.56 ^c^	0.69 ^c^	0.48 ^c^
* 8.65	476.2746 [M + Na]^+^	476.2748	−0.337	HMDB11503	LysoPE (16:0)	C_21_H_44_NO_7_P	Lipid metabolism	0.97 ^c^	1.34	1.43	1.69	1.98 ^c^	1.64	1.73	1.61
7.65	494.3246 [M + H]^+^	494.3241	0.980	HMDB10383	LysoPC (16:1)	C_24_H_48_NO_7_P	Lipid metabolism	1.64 ^c^	0.94	0.84 ^a^	0.86	0.82	0.66 ^b^	0.75 ^a^	0.68 ^b^
* 10.80	510.3913 [M + H]^+^	510.3918	−0.982	HMDB11149	LysoPC (O-18:0)	C_26_H_56_NO_6_P	Lipid metabolism	0.72 ^c^	0.92	0.94	0.97	0.87	0.84	0.78 ^a^	0.67 ^c^
* 7.84	520.3404 [M + H]^+^	520.3398	1.219	HMDB10386	LysoPC (18:2)	C_26_H_50_NO_7_P	Lipid metabolism	0.67 ^c^	0.87	1.01	1.00	0.98	1.09	0.99	0.89
* 9.05	522.3568 [M + H]^+^	522.3554	2.650	HMDB02815	LysoPC (18:1)	C_26_H_52_NO_7_P	Lipid metabolism	1.42 ^c^	1.02	0.90	0.92	0.86	0.71 ^b^	0.79 ^b^	0.74 ^b^
* 8.01	524.2754 [M + Na]^+^	524.2748	1.220	HMDB11517	LysoPE (20:4/0:0)	C_25_H_44_NO_7_P	Lipid metabolism	0.64 ^a^	1.38	1.67 ^a^	2.03 ^c^	1.91 ^c^	2.82 ^c^	2.26 ^c^	2.74 ^c^
* 7.49	540.3062 [M + Na]^+^	540.3061	0.258	HMDB10387	LysoPC (18:3)	C_26_H_48_NO_7_P	Lipid metabolism	0.97	1.37	1.43	1.41	1.40	1.79 ^b^	1.72 ^a^	1.59
* 7.76	548.2756 [M + Na]^+^	548.2748	1.532	HMDB11526	LysoPE (22:6/0:0)	C_27_H_44_NO_7_P	Lipid metabolism	1.03	1.23	1.43	1.48	1.05	2.22 ^c^	1.94 ^b^	1.90
* 8.34	568.3380 [M + Na]^+^	568.3374	1.125	HMDB10394	LysoPC (20:3)	C_28_H_52_NO_7_P	Lipid metabolism	0.67 ^c^	0.91	0.90	0.81	0.87	0.93	0.84	0.69
* 9.39	570.3533 [M + H]^+^	570.3554	−3.710	HMDB10402	LysoPC (22:5)	C_30_H_52_NO_7_P	Lipid metabolism	1.29 ^b^	0.96	0.91	0.90	0.79 ^a^	0.68 ^c^	0.75 ^b^	0.62 ^c^
* 7.22	282.2789 [M + H]^+^	282.2791	−0.855	HMDB02117	Oleamide	C_18_H_35_NO	Amino acid metabolism	0.65 ^c^	0.40 ^c^	0 ^c^	0 ^c^	0 ^c^	0 ^c^	0 ^c^	0 ^c^
2.91	132.0643 [M + H]^+^	132.0655	−9.236	HMDB02113	3-Hydroxy-l-proline	C_5_H_9_NO_3_	Amino acid metabolism	1.12	0.88	0.65	0.57 ^b^	0.82	0.91	1.03	0.94
* 8.16	301.2158 [M + Na]^+^	301.2138	6.635	HMDB01388	Alpha-Linolenic acid	C_18_H_30_O_2_	Lipid metabolism	0.42 ^c^	1.00	1.00	1.25	1.10	0.76	0.60	0.38 ^a^
* 8.84	303.2316 [M + H]^+^	303.2319	−0.847	HMDB01999	Eicosapentaenoic acid	C_20_H_30_O_2_	Lipid metabolism	0.51 ^c^	0.94	1.05	1.14	1.03	0.89	0.77	0.51
9.23	319.1939 [M + H]^+^	319.1904	11.010	HMDB06709	Ubiquinone-2	C_19_H_26_O_4_	Energy metabolism	0.36	0.45	1.20	1.19	9.15 ^b^	3.04	1.57	1.60
7.81	448.3413 [M + Na]^+^	448.3397	3.502	HMDB06464	Elaidic carnitine	C_25_H_47_NO_4_	Lipid metabolism	0.48 ^c^	1.15	1.28	1.28	1.20	1.34	0.71	1.09
7.29	372.3101 [M + H]^+^	372.3108	−1.975	HMDB05066	Tetradecanoyl carnitine	C_21_H_41_NO_4_	Lipid metabolism	0.49 ^c^	1.29	1.47	1.27	1.55	1.56	1.15	1.32
* 8.84	285.2208 [M + H]^+^	285.2213	−1.725	HMDB01358	Retinal	C_20_H_28_O	Lipid metabolism	0.52 ^c^	0.97	1.08	1.16	1.02	0.89	0.79	0.51 ^b^
* 10.32	283.2636 [M − H]^−^	283.2631568	1.565	HMDB00827	Stearic acid	C_18_H_36_O_2_	Lipid metabolism	0.92	0.93	1.02	1.11	1.15	1.30 ^b^	1.22 ^a^	1.23 ^a^
* 8.85	301.2167 [M − H]^−^	301.2162067	1.638	HMDB01999	Eicosapentaenoic acid	C_20_H_30_O_2_	Lipid metabolism	0.60 ^c^	0.92	1.03	1.10	0.93	0.82	0.72	0.49 ^c^
* 8.02	303.2323 [M − H]^−^	303.2318567	1.462	HMDB02177	*Cis*-8,11,14,17-Eicosatetraenoic acid	C_20_H_32_O_2_	Lipid metabolism	0.72 ^a^	1.14	1.47 ^b^	1.58 ^b^	1.66 ^c^	1.84 ^c^	1.61 ^c^	2.08 ^c^
9.39	319.2261 [M − H]^−^	319.2267713	−2.103	HMDB06245	18-Hydroxyarachidonic acid	C_20_H_32_O_3_	Lipid metabolism	0.07 ^c^	3.77	4.16	5.27	4.94	3.09	2.23	1.29
8.14	836.5423 [M − H]^−^	836.5436105	−1.567	HMDB10166	PS (18:0/22:5)	C_46_H_80_NO_10_P	Amino acid metabolism	0.41 ^c^	1.51	1.71 ^a^	1.49	1.37	0.73	0.72	0.35 ^a^
* 1.22	124.0071 [M − H]^−^	124.0062903	6.530	HMDB00251	Taurine	C_2_H_7_NO_3_S	Amino acid metabolism	0.48 ^c^	0.39	0.33	0.59	0.46	0.60	0.05 ^b^	0.19 ^a^
9.02	281.2481 [M − H]^−^	281.2475068	2.109	HMDB00207	Oleic acid	C_18_H_34_O_2_	Lipid metabolism	1.31 ^c^	0.96	0.96	0.99	1.02	0.91	0.96	0.95
* 8.74	255.2324 [M − H]^−^	255.2318567	2.129	HMDB00220	Palmitic acid	C_16_H_32_O_2_	Lipid metabolism	1.04	1.07	1.10	1.09	1.17	1.29 ^b^	1.23 ^a^	1.27 ^b^
* 7.30	227.2011 [M − H]^−^	227.2005566	2.392	HMDB00806	Myristic acid	C_14_H_28_O_2_	Lipid metabolism	1.57 ^c^	0.82 ^b^	0.78 ^b^	0.69 ^c^	0.61 ^c^	0.56 ^c^	0.73 ^c^	0.52 ^c^

RT: retention time; * had detected fragment ions listed in Table S1. C, control; M, model; US, United States; CA, Canada; HLJ, Heilongjiang; JL, Jilin; SD, Shandong; SX, Shanxi; BJ, Beijing; Fold change was calculated by the ratio of the average peak intensity of the latter group to the former group. a, b, c represented separately the significance *p* < 0.05, *p* < 0.01, and *p* < 0.001.

## Supplementary Materials

The following are available online. Figure S1: Typical total ion chromatograms of mouse serum sample analyzed by UPLC/Q-TOF-MS. Figure S2: OPLS-DA score plots of three groups of serum metabolomes. Table S1: The metabolites and their fragment ions detected by UPLC-Q/TOF-MS analysis.
