# Supplementary material for: Comparison of Serum Metabolite Changes of Radiated Mice Administered with Panax quinquefolium from Different Cultivation Regions Using UPLC-Q/TOF-MS Based Metabolomic Approach
Source: Molecules. 2018 Apr 26;23(5):1014. doi: 10.3390/molecules23051014 (PMC6102546; doi:10.3390/molecules23051014)
Supplement: Supplementary file 1 [file molecules-23-01014-s001.pdf]

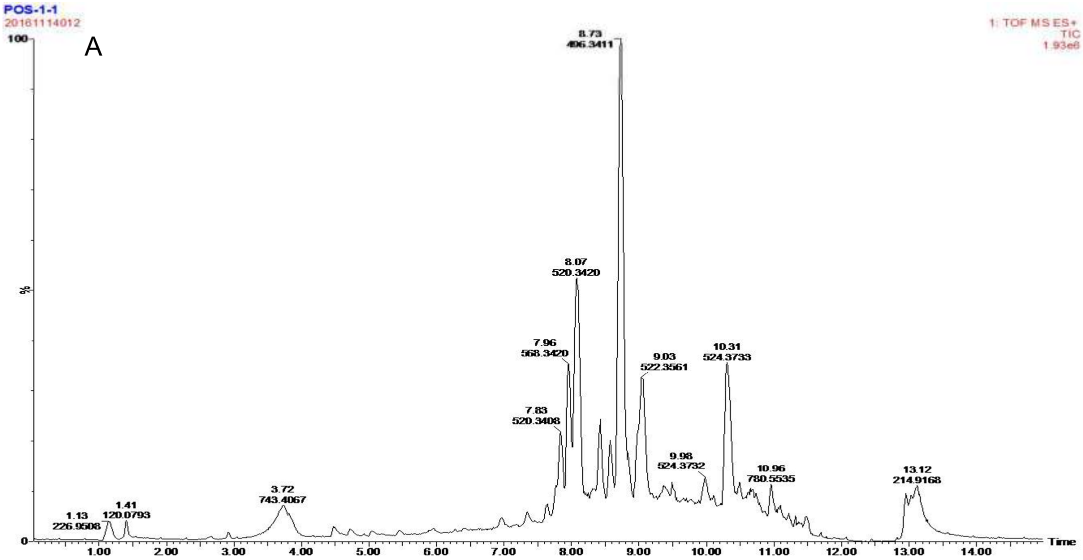

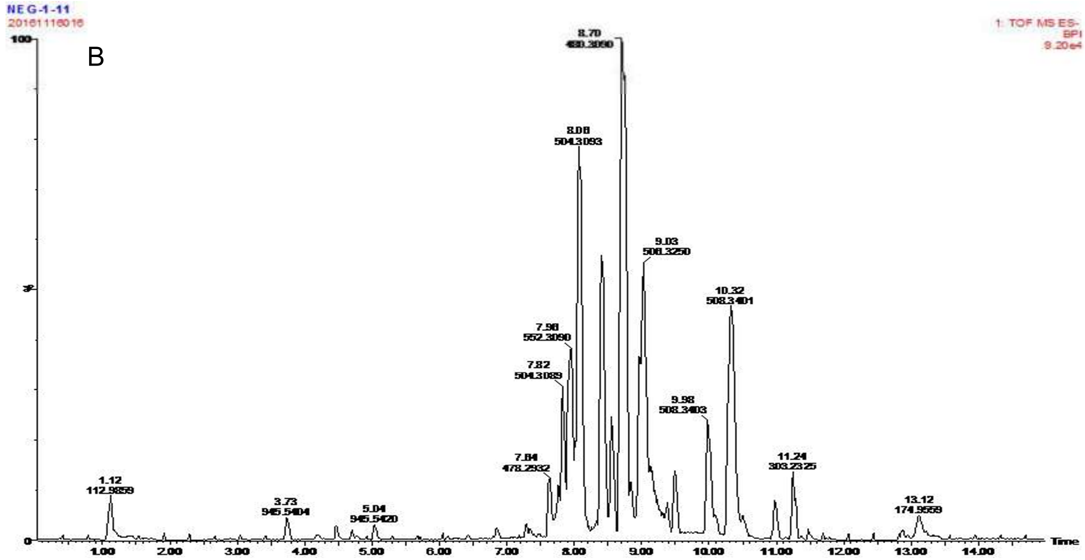

Figure S1. Typical total ion chromatograms of mouse serum sample analyzed by UPLC/Q-TOF-MS. (A) positive ESI mode and (B) negative ESI mode.

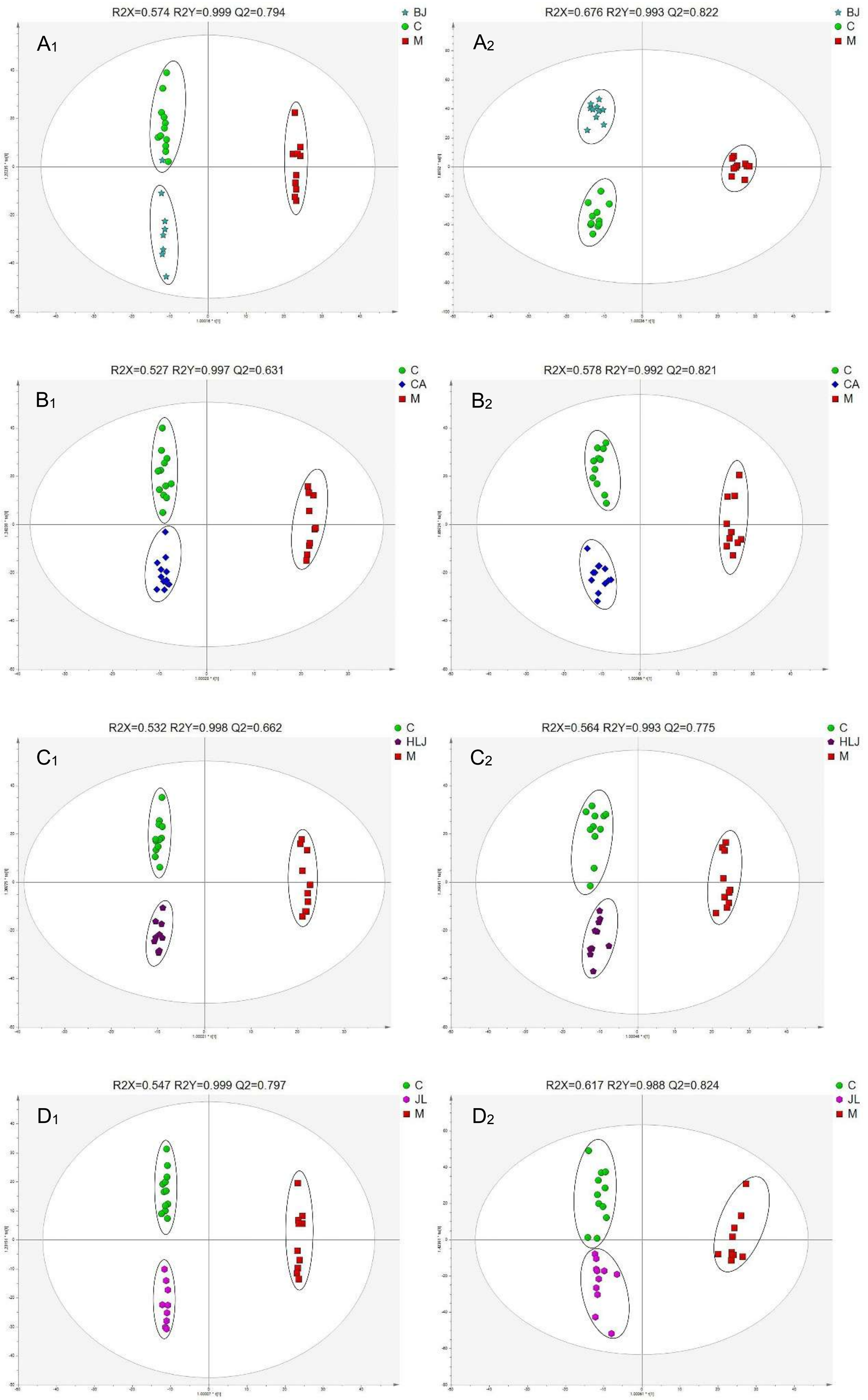
R2X=0.574 R2Y=0.999 Q2=0.794
A1
BJ
C
M
R2X=0.676 R2Y=0.993 Q2=0.822
A2
BJ
C
M
R2X=0.527 R2Y=0.997 Q2=0.631
B1
C
CA
M
R2X=0.578 R2Y=0.992 Q2=0.821
B2
C
CA
M
R2X=0.532 R2Y=0.998 Q2=0.662
C1
C
HLJ
M
R2X=0.564 R2Y=0.993 Q2=0.775
C2
C
HLJ
M
R2X=0.547 R2Y=0.999 Q2=0.797
D1
C
JL
M
R2X=0.617 R2Y=0.988 Q2=0.824
D2
C
JL
M

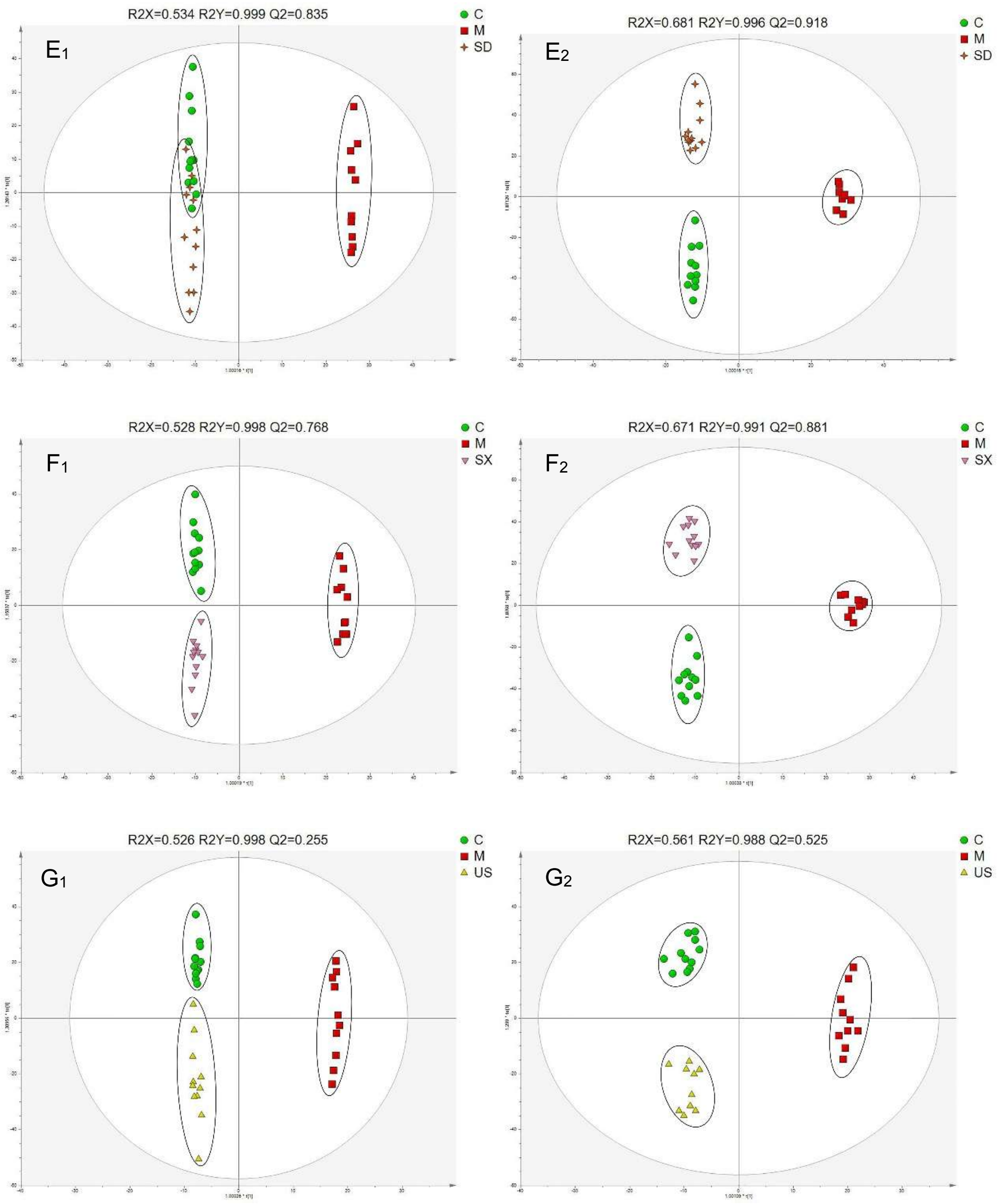

Figure S2. OPLS-DA score plots of three groups of serum metabolomes. ESI$^+$ mode: (A_1) to (G_1); ESI$^-$ mode: (A_2) to (G_2).

Table S1 The annotated metabolites and their fragment ions detected by UPLC-Q/TOF-MS analysis

RT_m/z	Identity	Ion Mode	Fragment ions detected
7.30_468.3084	LysoPC(14:0)	$[M+H]^+$	450.2968; 184.0736;104.1070
8.65_476.2746	LysoPE(16:0)	$[M+Na]^+$	458.7196; 415.2127; 335.0718
10.80_510.3913	LysoPC(O-18:0)	$[M+H]^+$	492.2522; 184.0724
7.84_520.3404	LysoPC(18:2)	$[M+H]^+$	502.3316; 184.0733; 104.1064
9.05_522.3568	LysoPC(18:1)	$[M+H]^+$	504.3450; 184.0727; 104.1071
8.01_524.2754	LysoPE(20:4/0:0)	$[M+Na]^+$	465.1195; 262.1111; 244.6862
7.49_540.3062	LysoPC(18:3)	$[M+Na]^+$	184.0732; 104.1062
7.76_548.2756	LysoPE(22:6/0:0)	$[M+Na]^+$	184.0732; 104.1062
8.34_568.3380	LysoPC(20:3)	$[M+Na]^+$	491.2267; 184.0722; 104.1063
9.39_570.3533	LysoPC(22:5)	$[M+H]^+$	184.0721; 104.1059
7.22_282.2789	Oleamide	$[M+H]^+$	59.0746; 72.9394
8.16_301.2158	Alpha-Linolenic acid	$[M+H]^+$	124.9979; 148.5315; 162.9452
8.84_303.2316	Eicosapentaenoic acid	$[M+H]^+$	285.2206; 258.6465
8.84_285.2208	Retinal	$[M+H]^+$	257.1878
10.32_283.2636	Stearic acid	[M-H]-	265.1415; 224.0720; 196.0360; 168.0441
8.85_301.2167	Eicosapentaenoic acid	$[M-H]^-$	257.2279
8.02_303.2323	Cis-8,11,14,17-Eicosatetraenoic acid	$[M-H]^-$	259.2415
1.22_124.0071	Taurine	$[M-H]^-$	106.9849; 60.7649
8.74_255.2324	Palmitic acid	$[M-H]^-$	237.2283; 211.7911
7.30_227.2011	Myristic acid	$[M-H]^-$	182.9917; 168.9812; 154.1260

Literatures for fragment ions identified

1. Xu X.B.; Gao B.B.; Guan Q.J.; Zhang D.D.; Ye X.H.; Zhou L.; Tong G.X.; Li H.; Zhang L.; Tian J.K.; Huang J.Y. Metabolomic profile for the early detection of coronary artery disease by using UPLC-QTOF/MS. J Pharm Biomed Anal **2016**, 129:34-42.

2. Ding M.J.; Zong Z.M.; Zong Y.; Ou-Yang X.D.; Huang Y.G.; Zhou L.; Wang F.; Cao J.P.; Wei X.Y. Isolation and identification of fatty acid amides from shengli coal. Energy Fuels **2008**, 22(4):2419-2421.

3. Du L.N. Research of metabolic characteristic of Respiratory syncytial virus pneumonia with syndrome of obstruction of the lung by phlegm heat based on UPLC-MS technology and evaluation of JinXin Oral Liquid [D]. Nanjing University of Chinese Medicine, 2015.

4. Jin Z.N. The hypolipidaemic effect of *Curcuma longa* L. and the full characterization and separation of curcuminoids based on a combinatorial LC-MS/MS technologies [D]. Huazhong University of Science and Technology, 2015.
